# Hip Accelerometry Activity Patterns Improve Machine Learning Prediction of 1-Year MoCA Score Change

**DOI:** 10.1093/geroni/igab046.1723

**Published:** 2021-12-17

**Authors:** Chengjian Shi, Jacek Urbanek, Niser Babiker, Alan Gonzolez, Jovany Soto, Andrey Rzhestsky, Megan Huisingh-Scheetz

**Affiliations:** 1 University of Chicago, chicago, Illinois, United States; 2 Johns Hopkins School of Medicine, Baltimore, Maryland, United States; 3 University of Chicago, Chicago, Illinois, United States; 4 Illinois Institute of Technology, Orland Park, Illinois, United States

## Abstract

We tested whether free-living hip accelerometry measures improved prediction of 1-year change in Montreal Cognitive Assessment (MoCA) scores beyond clinically available information. We analyzed data (n=126) from predominantly African American (78.2%) older adults without moderate-severe dementia residing near our geriatrics clinic. Age (73.6 ±6.1 years), gender, education, comorbidities, income, and MoCA performance were collected at baseline; participants then wore a right hip, triaxial Actigraph accelerometer (30Hz) continuously for 7 days. A MoCA was repeated at 1 year. Six measures were calculated from the daytime (7am-5pm) data: mean/variance of hourly counts per minute, mean/variance of daily percent of time spent in the lowest activity quartile, and mean/variance of daily percent of time spent in the highest activity quartile. In a random forest model containing baseline MoCA, demographics and comorbidities, the accelerometry measures improved prediction of 1-year MoCA performance by ~17.8%. Accelerometry data may be clinically useful for predicting early cognitive decline.

